# Stem Bark Extract and Fraction of *Persea americana* (Mill.) Exhibits Bactericidal Activities against Strains of *Bacillus cereus* Associated with Food Poisoning

**DOI:** 10.3390/molecules20010416

**Published:** 2014-12-30

**Authors:** David A. Akinpelu, Olayinka A. Aiyegoro, Oluseun F. Akinpelu, Anthony I. Okoh

**Affiliations:** 1 SA-MRC Microbial Water Quality Monitoring Centre, University of Fort Hare, Alice 5700, South Africa; E-Mails: dakinpel@yahoo.com (D.A.A.); aokoh@ufh.ac.za (A.I.O.); 2 Applied and Environmental Microbiology Research Group, Department of Biochemistry and Microbiology, University of Fort Hare, Alice 5700, South Africa; 3 Department of Microbiology, Obafemi Awolowo University, Ile Ife, Osun State 234, Nigeria; 4 GI Microbiology and Biotechnology Unit, Agricultural Research Council, Animal Production Institute, Irene, Pretoria 0062, South Africa; 5 Department of Biological Science, Faculty of Agriculture and Technology, North West University, Mafikeng Campus, Mmabatho 2735, South Africa; E-Mail: Sewizzy2009@yahoo.com

**Keywords:** *Persea americana*, *Bacillus cereus*, antimicrobial activity, killing rate, protein leakage, potassium ion leakage, minimum inhibitory concentration

## Abstract

The study investigates the *in vitro* antibacterial potentials of stem bark extracts of *Persea americana* on strains of *Bacillus cereus* implicated in food poisoning. The crude stem bark extracts and butanolic fraction at a concentration of 25 mg/mL and 10 mg/mL, respectively, exhibited antibacterial activities against test isolates. The zones of inhibition exhibited by the crude extract and the fraction ranged between 10 mm and 26 mm, while the minimum inhibitory concentration values ranged between 0.78 and 5.00 mg/mL. The minimum bactericidal concentrations ranged between 3.12 mg/mL–12.5 mg/mL and 1.25–10 mg/mL for the extract and the fraction, respectively. The butanolic fraction killed 91.49% of the test isolates at a concentration of 2× MIC after 60 min of contact time, while a 100% killing was achieved after the test bacterial cells were exposed to the butanolic fraction at a concentration of 3× MIC after 90 min contact time. Intracellular protein and potassium ion leaked out of the test bacterial cells when exposed to certain concentrations of the fraction; this is an indication of bacterial cell wall disruptions by the extract’s butanolic fraction and, thus, caused a biocidal effect on the cells, as evident in the killing rate test results.

## 1. Introduction

Plant products play an important role in healthcare delivery as therapeutic remedies in the world, especially in developing countries. This has caused phytomedicine to become an integral part of the healthcare system of many nations. Medicinal plants are rich in bio-resources of drugs, and these chemical compounds play a definite physiological action in the human body system [1]. Investigation of medicinal plants by ethno-botanists as an alternative to the existing synthetic medicines has been on the increase; this is because of the fact that most man-made medications are now progressively losing their potency to pathogens. In addition to the multiple resistance developed by pathogens against the synthetic antibiotics, most of these drugs have a major setback due to the side effects on the patients. Medicinal plants are likely to be more tolerated by humans than the synthetic antimicrobial agents, because pants are products of nature.

Foodborne illnesses caused by pathogens are a wide-spread public health problem globally. *Bacillus cereus*, a Gram-positive bacteria, is known for its ability to cause food poisoning [2]. Food poisoning is one of the leading causes of illness and death in developing countries, and one-third of the population is estimated to be affected by microbiological foodborne diseases each year [3]. *Bacillus cereus* is found in a wide range of habitats, such as air, water, soil and raw food, such as potatoes, beans, spices and rice [4]. Turbull and co-workers [5] reported that this organism has gained resistance to most antibiotics, especially penicillin, cephalosporin and trimethoprim. These have posed a threat to public health, and there is a need to source for more potent antimicrobial agents to stop the spread of this organism and other pathogens that are now gradually developing resistance to the available synthetic antimicrobial drugs.

*Persea americana* belongs to the family Lauraceae. The English name is avocado, and it is widely cultivated in tropical and subtropical regions [6]. The fruits are loaded with nutrients, such as vitamin E, potassium, magnesium, vitamin B and K and monosaturated fatty acids [7]. The plant is used in the management of hypertension [8]; while the carotenoid content of the edible fruit pulp may play a significant role in cancer reduction [9]. The seeds of *P. americana* are used in the treatment of diarrhea, dysentery, toothache and skin infections [10]. *Persea americana* leaf extract possesses anti-inflammatory and analgesic effects [11] and was also reported to possess antifungal properties [12]. The aqueous stem bark extract of the plant is used traditionally for the treatment of skin infection [13], while the seeds are used to treat asthma, high blood pressure and rheumatism among the Yoruba tribe of South Western Nigeria.

## 2. Results

Five fractions were obtained from the crude stem bark extract of *Persea americana*, and these include aqueous (AQ), n-butanol (BL), chloroform (CL), ethyl acetate (EA) and n-hexane (HX) fractions. Only the n-butanol fraction exhibited appreciable antibacterial activities at a concentration of 10 mg/mL against all of the thirty-three strains of *Bacillus cereus* obtained from various sources. The aqueous fraction exhibited antibacterial activity against three out of the thirty-three test isolates, while the ethyl acetate and n-hexane fractions exhibited antibacterial activities against one of each of the test isolates. The two standard antibiotics, ampicillin and streptomycin, each at a concentration of 1 mg/mL, exhibited antibacterial activities against all of the test isolates (Table 1).

**Table 1:** Sensitivity patterns of zones of inhibition exhibited by the fraction obtained from the *Persea americana* extract on bacterial isolates.

Bacterial Codes	Zones of Inhibition (mm) **				
	BL (10 mg/mL)	AQ (10 mg/mL)	HX (10 mg/mL)	CL (10 mg/mL)	EA (10 mg/mL)
B1	20 ± 0.94	0	0	0	0
B2	19 ± 0.82	0	0	0	0
B3	22 ± 0.00	0	12.67 ± 0.94	0	14.67 ± 0.94
B4	17 ± 0.62	0	0	0	0
B5	24 ± 0.94	00 ± 0.00	0	0	0
B6	12 ± 0.00	0	0	0	0
B7	13 ± 0.24	11.67 ± 0.47	0	0	0
B8	16 ± 0.41	10.67 ± 0.94	0	0	0
B9	17 ± 0.82	0	0	0	0
B10	16 ± 0.24	0	0	0	0
B11	16 ± 0.00	0	0	0	0
B12	18 ± 0.47	0	0	0	0
B13	14 ± 0.62	0	0	0	0
B14	19 ± 0.82	0	0	0	0
B15	14 ± 0.00	0	0	0	0
B16	18 ± 0.00	0	0	0	0
B17	24 ± 0.94	0	0	0	0
B18	25 ± 0.94	0	0	0	0
B19	14 ± 0.00	0	0	0	0
B20	15 ± 0.94	0	0	0	0
B21	22 ± 0.00	10.67 ± 0.94	0	0	0
B22	25 ± 0.47	0	0	0	0
B23	23 ± 0.94	0	0	0	0
B24	19 ± 0.82	0	0	0	0
B25	23 ± 0.82	0	0	0	0
B26	16 ± 0.00	0	0	0	0
B27	26 ± 0.47	0	0	0	0
B28	16 ± 0.94	0	0	0	0
B29	18 ± 0.94	0	0	0	0
B30	23 ± 0.47	0	0	0	0
B31	22 ± 0.47	0	0	0	0
B32	17 ± 0.82	0	0	0	0
B33	20 ± 0.00	0	0	0	0

Key: B1–B10 = *Bacillus cereus* (environmental strains); B11–B20 = *Bacillus cereus* (clinical strains); B21–B31 = *Bacillus cereus* (vomitus strains); B32 = *Bacillus cereus* NCIB 6349; B33 = *Bacillus cereus* ATCC 14579; **, mean of three replicates; BL = butanol fraction; AQ = aqueous fraction; HX = N-hexane fraction; CL = chloroform fraction; EA = ethyl–acetate fraction.

The crude stem bark extract of the plant tested at a concentration of 25 mg/mL inhibited the growth of all of the test isolates (Table 2).

**Table 2:** Susceptibility patterns exhibited by the crude extract of *Persea americana* and the standard antibiotics against the test bacterial isolates.

Bacterial Code	Zones of Inhibition (mm) **		
	Crude Extract (25 mg/mL)	Streptomycin (1 mg/mL)	Ampicillin (1 mg/mL)
B1	22 ± 0.41	23 ± 0.47	22 ± 0.00
B2	17 ± 0.47	22 ± 0.00	25 ± 0.94
B3	15 ± 0.94	20 ± 0.47	26 ± 0.00
B4	16 ± 0.94	24 ± 0.94	28 ± 0.00
B5	10 ± 0.47	28 ± 0.00	26 ± 0.00
B6	11 ± 0.94	27 ± 0.82	24 ± 0.00
B7	13 ± 0.82	20 ± 0.94	24 ± 0.00
B8	11 ± 0.47	20 ± 0.47	24 ± 0.82
B9	16 ± 0.00	26 ± 0.00	25 ± 0.82
B10	13 ± 0.94	24 ± 0.00	24 ± 0.00
B11	17 ± 0.94	28 ± 0.00	23 ± 0.82
B12	16 ± 0.00	25 ± 0.47	20 ± 0.00
B13	15 ± 0.47	25 ± 0.47	22 ± 0.94
B14	11 ± 0.41	22 ± 0.94	28 ± 0.00
B15	15 ± 0.47	23 ± 0.47	22 ± 0.00
B16	12 ± 0.00	23 ± 0.94	26 ± 0.00
B17	17 ± 0.94	26 ± 0.00	24 ± 0.00
B18	12 ± 0.00	27 ± 0.82	22 ± 0.00
B19	16 ± 0.94	26 ± 0.00	21 ± 0.47
B20	14 ± 0.00	22 ± 0.00	24 ± 0.00
B21	17 ± 0.94	25 ± 0.94	26 ± 0.00
B22	16 ± 0.94	25 ± 0.94	24 ± 0.00
B23	17 ± 0.47	24 ± 0.00	26 ± 0.94
B24	15 ± 0.82	26 ± 0.00	22 ± 0.00
B25	13 ± 0.94	24 ± 0.00	24 ± 0.00
B26	14 ± 0.00	22 ± 0.94	26 ± 0.00
B27	13 ± 0.47	25 ± 0.82	23 ± 0.47
B28	13 ± 0.94	26 ± 0.00	26 ± 0.00
B29	11 ± 0.82	25 ± 0.94	24 ± 0.00
B30	18 ± 0.94	23 ± 0.94	25 ± 0.47
B31	15 ± 0.94	25 ± 0.94	26 ± 0.00
B32	19 ± 0.94	22 ± 0.00	26 ± 0.00
B33	17 ± 0.82	24 ± 0.00	26 ± 0.00

Key: B1–B10 = *Bacillus cereus* (environmental strains); B11–B20 = *Bacillus cereus* (clinical strains); B21-B31 = *Bacillus cereus* (vomitus strains); B32 = *Bacillus cereus* NCIB 6349; B33 = *Bacillus cereus* ATCC 14579; **, mean of three replicates.

The zones of inhibition exhibited by the butanolic fraction ranged between 12 mm and 26 mm, while the zones of inhibition shown by the aqueous fraction against the three isolates ranged between 10 mm and 11 mm. On the other hand, the zones of inhibition exhibited by the crude extract were between 10 mm and 22 mm. The zones of inhibition exhibited by ampicillin and streptomycin used as a positive control ranged between 20 mm and 28 mm. The aqueous, ethyl acetate and n-hexane fractions showed limited activities against the test isolates and, thus, were not used for further tests. The antimicrobial activity exhibited by the butanolic fraction compared favorably with those of the two standard antibiotics, ampicillin and streptomycin, used in this study.

The MIC and minimum bactericidal concentration (MBC) of the crude extract, butanolic fraction and those of ampicillin and streptomycin were determined. The MIC of the crude extract exhibited against the test isolates ranged between 0.78 mg/mL and 12.5 mg/mL, while those of the butanolic fraction were between 0.63 mg/mL and 5.00 mg/mL. The minimum bactericidal effects of the crude extract showed against the test isolates were between 3.12 mg/mL and 12.5 mg/mL. On the other hand, the MBC exhibited by the butanolic fraction against the test isolates ranged between 1.25 mg/mL and 10.0 mg/mL. Ampicillin and streptomycin exhibited MIC ranging between 0.03 mg/mL and 0.25 mg/mL and 0.03 mg/mL and 0.13 mg/mL, respectively. The MBCs exhibited by ampicillin against the organisms were between 0.06 mg/mL and 0.50 mg/mL, while the same range was also exhibited by streptomycin (Table 3). From all indications, the two standard antibiotics showed better activities against the test isolates than the butanolic fraction.

**Table 3:** The minimum inhibitory concentrations of the crude extract, butanol fraction and standard antibiotics exhibited against susceptible bacterial isolates.

Bacterial Code	Crude Extract (25 mg/mL)		Butanol Fraction (10 mg/mL)		Streptomycin (1 mg/mL)		Ampicillin (1 mg/mL)	
	MIC (mg/mL)	MBC (mg/mL)	MIC (mg/mL)	MBC (mg/mL)	MIC (mg/mL)	MBC (mg/mL)	MIC (mg/mL)	MBC (mg/mL)
B1	3.12	6.25	1.25	2.5	0.06	0.12	0.25	0.50
B2	1.56	3.12	0.63	1.25	0.06	0.25	0.03	0.06
B3	1.56	3.12	2.50	5.00	0.12	0.25	0.12	0.12
B4	3.12	6.25	2.50	5.00	0.03	0.06	0.06	0.25
B5	0.78	3.12	0.63	1.25	0.06	0.12	0.25	0.50
B6	6.25	12.50	0.63	1.25	0.06	0.12	0.06	0.12
B7	6.25	6.25	0.62	2.50	0.25	0.25	0.12	0.25
B8	1.56	6.25	2.50	2.50	0.03	0.12	0.12	0.25
B9	3.12	6.25	1.25	1.25	0.03	0.12	0.25	0.50
B10	6.25	12.50	0.62	1.25	0.25	0.50	0.06	0.12
B11	6.25	6.25	1.25	5.00	0.25	0.50	0.06	0.12
B12	6.25	6.25	0.62	1.25	0.06	0.12	0.12	0.25
B13	12.50	12.50	10.00	10.00	0.25	0.50	0.06	0.12
B14	3.12	6.25	2.50	5.00	0.25	0.50	0.06	0.12
B15	3.12	12.50	0.63	2.50	0.12	0.50	0.03	0.06
B16	1.56	3.12	5.00	5.00	0.06	0.25	0.12	0.25
B17	1.56	3.12	1.25	2.50	0.12	0.25	0.12	0.25
B18	6.25	6.25	1.25	2.50	0.12	0.25	0.12	0.25
B19	6.25	12.50	10.00	10.00	0.06	0.12	0.06	0.12
B20	1.56	3.12	1.25	2.50	0.12	0.25	0.12	0.25
B21	6.25	6.25	1.25	2.50	0.12	0.50	0.12	0.12
B22	6.25	12.50	2.50	5.00	0.12	0.25	0.06	0.25
B23	1.56	3.12	0.63	1.25	0.31	0.12	0.12	0.25
B24	3.12	6.25	5.00	10.00	0.25	0.50	0.06	0.12
B25	12.5	12.50	1.25	2.50	0.06	0.25	0.25	0.50
B26	6.25	12.50	10.00	10.00	0.25	0.25	0.06	0.25
B27	3.12	6.25	1.25	2.50	0.12	0.25	0.25	0.25
B28	0.78	3.12	1.25	5.00	0.12	0.50	0.03	0.12
B29	6.25	6.25	0.63	1.25	0.06	0.12	0.06	0.12
B30	1.56	6.25	2.50	2.50	0.25	0.25	0.06	0.12
B31	6.25	6.25	1.25	2.50	0.12	0.12	0.03	0.12
B32	3.12	6.25	2.50	2.50	0.25	0.50	0.12	0.12
B33	6.25	12.50	1.25	5.00	0.25	0.50	0.06	0.12

Key: B1–B10 = *Bacillus cereus* (environmental strains); B11–B20 = *Bacillus cereus* (clinical strains); B21–B31 = *Bacillus cereus* (vomitus strains); B32 = *Bacillus cereus* NCIB 6349; B33 = *Bacillus cereus* ATCC 14579; MIC = minimum inhibitory concentration; MBC = minimum bactericidal concentration.

The phytochemical components of the extract were investigated. The extract revealed the presence of alkaloids, tannins, saponins, flavonoids and reducing sugars (Table 4).

**Table 4:** Phytochemical compounds present in the stem bark extract of *Persea americana.*

Chemical Test	Result
Alkaloids	Positive
Tannins	Positive
Saponins	Positive
Flavonoid	Positive
Reducing sugar	Positive

**Figure 1:**
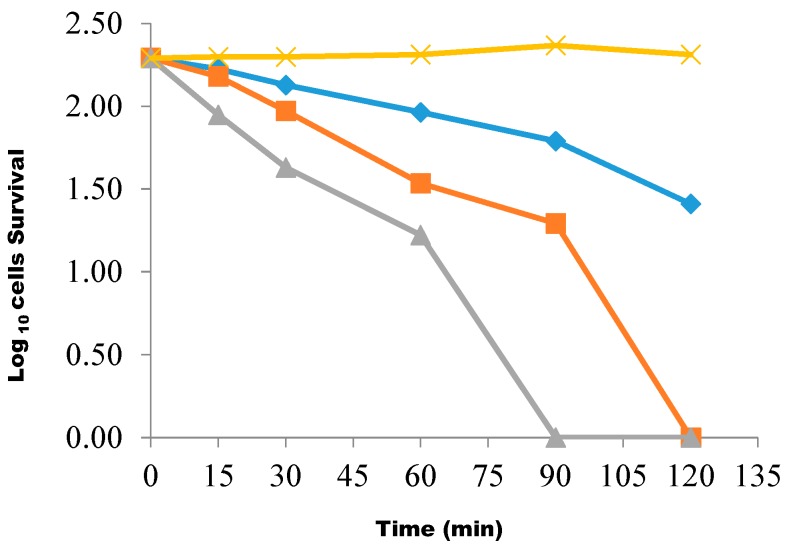
The extent and the rate of killing of test bacterial cells by the butanol fraction at concentrations of 1× MIC (◊), 2× MIC (■), 3× MIC (▲) and the control (X). Each point represents the log_10_ of the mean survival of bacterial cells at a particular time interval in the presence of the fraction.

The killing rate, protein and potassium ion leakages of the effects of butanolic fraction on the test isolates were also determined for bactericidal efficacy. Figure 1 revealed the extent and killing rate of the test isolate by butanolic fraction at a concentration of 1× MIC. Within a 15-min contact time interval between the fraction suspension and the test isolates, the percentage of the bacterial cells killed was 13.8%, while this rose to 31.9% after 30 min of reaction. At a 60 min contact time, the percentage of the cells killed was 52.8%, and after 90 min of reaction time, 86.4% of the cells were killed. Overall, after the reaction was stopped at 120 min of reaction time, about 90% of the cell suspension was killed. This monophasic trend was also observed when the concentration of the extract doubled, that is 2× MIC. The concentration was again increased to 3× MIC, and the percentage of the cells killed after a 15-min time contact interval was 54.9%. When the contact time was increased to 30 and 60 min, the percentage of the cells killed was 78.1% and 91.49%, respectively. Finally, 100% cells died after a 90 min contact time interval (Figure 1).

**Figure 2:**
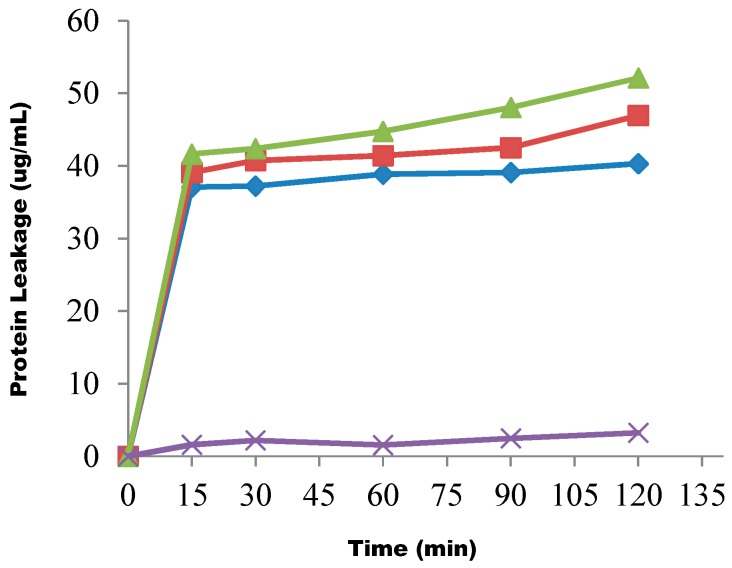
The effect of the butanol fraction on protein leakage from test cells at concentrations of 1× MIC (◊), 2× MIC (■), 3× MIC (▲) and control (X). Each point represents the amount of protein leaked (µg/mL) from the cells at a particular time interval in the presence of the fraction.

The biocidal effect of the fraction on the cells was also observed through cell membrane disruption, leading to protein leakage from the test cells, as shown in Figure 2.

At a 1× MIC concentration and 15-min contact time of the cells with the fraction suspension, about 37.0 µg/mL of protein leaked out of the test cells. When the timing was increased to 30 min, 37.8 µg/mL of protein was released from the test cells. At 60, 90 and 120 min contact time intervals, 38.9 µg/mL, 39.1 µg/mL and 40.3 µg/mL of protein leaked out of the cells, respectively. This same trend of protein leakage was observed when the fraction concentration was increased to a 2× MIC rate. The fraction concentration was later increased to 3× MIC, and the test cells were subjected to the effect of the drug. First, at a 15 min contact time of cells with the suspension, 41.6 µg/mL of protein leaked out of the cells, while this increased to 42.4 µg/mL after a 30 min contact time. At contact times of 60 and 90 min, the quantity of protein leaked out of the cells increased to 44.7 µg/mL and 48.3 µg/mL, respectively. Finally, the percentage of protein leaking out of the cells arrived at 57.1 µg/mL after 120 min. A monophasic effect was also observed in this test.

**Figure 3:**
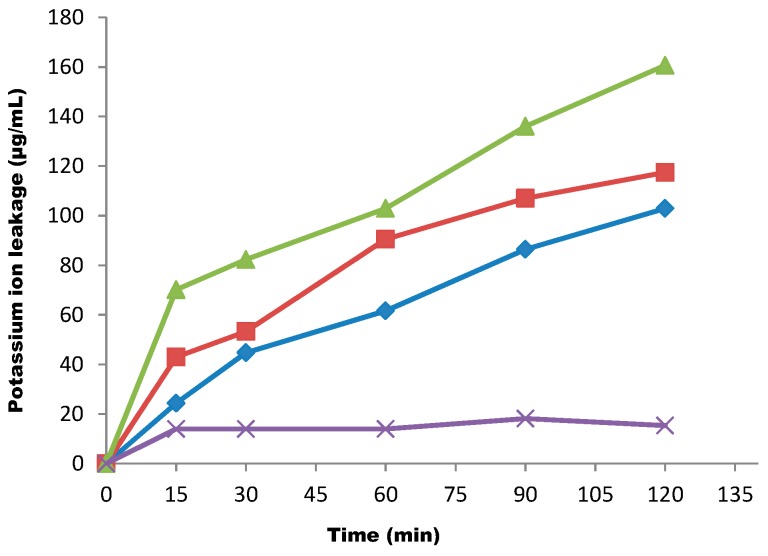
The effect of the butanol fraction on potassium ion leakage from test cells at concentrations of 1× MIC (◊), 2× MIC (■), 3× MIC (▲) and control (X). Each point represents the amount of potassium ions leaked (µg/mL) from the cells at a particular time interval in the presence of the fraction.

Potassium ion leakage from the cells was also studied using different concentrations of the fraction over a determined period of time (Figure 3).

When the cells were subjected to the effect of the suspension of the fraction over a period of 15 min at a concentration of 1× MIC, about 24.3 µg/mL of potassium ions leaked out of the cells. At 30, 60 and 90 min contact times of the cells with the suspension of the fraction, 44.7 µg/mL, 61.5 µg/mL and 86.4 µg/mL, respectively, of potassium ions leaked out of the test cells. Finally, at a 120 min contact time, the concentration of potassium ion leaked out of the cells was 102.93 µg/mL. The same trend of leakage was observed when the concentration of the fraction was increased to 2× MIC. The test cells were again treated with a high concentration of 3× MIC of the suspension. At a contact time of 120 min, the quantity of potassium ions leaked out of the cells was 160.7 µg/mL (Figure 3).

## 3. Discussion

The antimicrobial potentials of stem bark extracts of *Persea americana* were investigated against a panel of *Bacillus cereus* strains obtained from various sources. This pathogen is known for its roles in food poisoning. Among the fractions obtained from the crude extract of *P*. *americana*, the butanolic fraction exhibited appreciable antimicrobial activities against all of the test isolates at a concentration of 10 mg/mL. Other fractions, that is aqueous, ethyl acetate, chloroform and n-hexane, showed minimal or no antimicrobial activity. Among the solvents used in partitioning the extract, butanol has the highest polarity, and its fraction exhibited the highest activity, thus an indication that the bioactive components of *P. americana* have more affinity for a polar solvent. This might serve to point toward the type of organic solvent to be used in extracting the active components of this plant. When comparing the antimicrobial activity exhibited by the butanolic fraction with those of the standard antibiotics—ampicillin and streptomycin; used in this study, the fraction compared favorably with the two positive controls. Ampicillin and streptomycin exhibited lower MIC and MBC than the butanolic fraction; this is considering the fact that these antibiotics are in a pure form, but the fraction is still partially purified. If this fraction were purified, it would compete better with these antibiotics and, thus, can serve as a source of potent drug. The phytochemical screening of the stem bark extracts of *P. americana* revealed the presence of alkaloids, flavonoids, saponins, reducing sugars and tannins. These compounds are known to be biologically active and contribute to the antimicrobial and antioxidant activities of medicinal plants [14] and, thus, contributed to the bioactive activity of *P. americana*. The presence of these compounds in *P*. *americana* supported its usefulness in folklore remedies. Phytochemicals have been reported to cause the leakage of intracellular materials from cells as a result of the disruption of the cell membrane [15]. From our observations, *P*. *americana* exerted a biocidal effect on the test cells, and thus, these phytochemicals might also be involved in this activity. Our findings on the kill rate exhibited by the plant extract showed a 100% kill of the test cells at a 3× MIC within 90 min of the cells’ interaction with the suspension of the extract. Total killing of organisms by antimicrobial agents within the shortest time is an indication of a better bactericidal effect by such a compound [16]. Thus, the bactericidal effects exhibited by *P*. *americana* stem bark extract observed in our investigation showed a significant therapeutic potential of this plant and, thus, support its usefulness in folklore remedies in combating the activity of pathogens responsible for various diseases, including foodborne illness. The leakage of proteins and potassium ions from cells of *B. cereus* was investigated in our studies. The results observed showed appreciable leakage of these protoplasmic materials, which may actually lead to the death of such organism (Figure 2 and Figure 3). In addition, the leakage of materials from the cells as observed in our findings indicate cell membrane disruption caused by *P*. *americana* stem bark extract. An increase in the permeability of the cytoplasmic membrane will lead to the loss of these cellular matters and, consequently, results in cell death [17]. Sanati and co-workers [18] observed such an occurrence when investigating the action of triazole voriconazole on *Candida albicans*. The ability of *P*. *americana* extract to kill the test isolate used in this study and known to be pathogen at minimal contact time and a low concentration could be used in preventing the establishment of infections caused by this pathogen. Our observations in this study proved the use of *Persea americana* in folklore remedies for the treatment of various diseases caused by microorganisms.

## 4. Experimental Section

### 4.1. Plant Materials

Fresh stem bark of *Persea americana* was collected in Ile Ife, Osun State, Nigeria, in the month of October, 2013. The stem bark was identified in the Herbarium of the Department of Botany, Obafemi Awolowo University, Ile Ife, Nigeria. The sample was cut into pieces and dried in a hot-air oven at 40 °C to constant weight, powdered and kept in an air-tight container for further use.

### 4.2. Preparation of the Plant Extract

Exactly 1.5 kg of the powdered plant sample was soaked in a mixture of methanol and sterile distilled water in a ratio of 3:2 (*v*/*v*) for four days with regular agitation periodically. The supernatant was filtered into a clean flask and later concentrated *in vacuo* using a rotary evaporator to eliminate the methanol. The aqueous residue left was then lyophilized to obtain a 0.16 kg yield of a light brown crude extract.

### 4.3. Preparation of Test Bacterial Isolates

The typed cultures of the National Collection of Industrial Bacteriology (NCIB) and the American Typed Culture Collection (ATCC) along with the environmental strains (ES), isolated from fruits and vegetables, soil, water, vomitus strains (VS), isolated from the vomit of sick patients, and clinical isolates (CI), isolated from clinical samples, e.g., pus, sputum, blood, *etc.*, of *Bacillus cereus* were used in this study. These isolates were first standardized (10^6^ cfu/mL), then sub-cultured in nutrient broth (Mast Group, Bootle, UK) and incubated at 37 °C for 18 h before use.

### 4.4. Culture Media Used

Nutrient agar medium (Mast Group, Bootle, UK) was used for sub-culturing the test organisms, while Mueller-Hinton agar medium (Mast Group, Bootle, UK) was used for the sensitivity testing.

### 4.5. Phytochemical Assays

The crude extract was subjected to phytochemical tests using the Trease and Evans and the Harborne [14,19] methods to test for the bioactive components in the plant sample, which include the test for alkaloids, tannins, saponins, flavonoids and reducing sugars.

### 4.6. Fractionation of Crude Extract of the Plant Sample

The crude extract was successfully partitioned using organic solvents, which include n-hexane, chloroform, ethyl acetate and n-butanol, in order of their polarity. One hundred and five grams of the stem bark extract of the plant sample was resolved in 200 mL of sterile distilled water in a separatory funnel and extracted with *n*-hexane (4 × 200 mL). The resulting n-hexane phase was concentrated to dryness *in vacuo*, and the residue (15.5 g) was kept in a freezer (−4 °C) in an air-tight container for further use. The resulting aqueous phase was re-concentrated to remove traces of n-hexane. The residue collected was further extracted with chloroform (4 × 200 mL). The chloroform fraction collected was also concentrated *in vacuo* to dryness, and a 12.8 g yield of the fraction was collected. This was also kept in a freezer (−4 °C) for further use. The ethyl acetate (15.2 g) and butanol (30.6 g) fractions were also obtained using similar procedure. The remaining aqueous fraction was freeze-dried to yield a 27.2 g fraction and kept in a freezer (−4 °C) for further use.

### 4.7. The Antibiograms of P. americana Crude Stem Bark Extract, Fractions and the Standard Antibiotics—Streptomycin and Ampicillin—Against Test Isolates

The sensitivity testing of the crude extract of *P. americana* along with those of the standard antibiotics—ampicillin and streptomycin—was determined using the agar-well diffusion method described by Russell and Furr and Irobi [20,21] with little modification. The bacterial isolates were first grown in nutrient broth to re-activate the cells. The broth culture was later standardized to 10^6^ cfu/mL of 0.5 McFarland standard. One hundred microliters of the standardized bacterial suspension were evenly spread on Mueller-Hinton agar medium using a sterile glass spreader. With the aids of a sterile 6 mm cork borer, wells were bored into the agar medium allowing about a 5 mm distance to the edge of the plate. The wells were then filled-up (1 mL) with the solution of the crude extract at a concentration of 25 mg/mL. Care was taken not to allow spillage of the solution on the surface of the agar medium. The plates were allowed to stand on the laboratory bench for 1 h to allow proper diffusion of the extract into the medium. The plates were later incubated in an upright position in the incubator at 37 °C for 24 h, after which they were examined for zones of inhibition. The effects of the extract on the test isolates were compared with those of ampicillin and streptomycin, used as positive controls.

### 4.8. Determination of Minimum Inhibitory Concentrations (MICs) of the Extract and Fractions of P. americana

The MICs of the crude extract and partitioned fractions were determined using two-fold dilutions of the extracts as described by Akinpelu and Kolawole [22]. Two-fold dilution of the extract was prepared, and 2 mL of different concentrations of the extract solution were added to 18 mL of pre-sterilized molten nutrient agar after it cooled down to 40 °C to give final concentration regimes of 0.391 to 25.0 mg/mL for the crude extract and 0.625 to 10.0 mg/mL for the fraction. The medium was then poured into Petri dishes and allowed to set. The plates were left on the laboratory bench for 24 h to observe their sterility. The dry surface of the media was later streaked with 18 h-old test isolates. The plates were incubated at 37 °C for up to 72 h, after which they were examined for the presence or absence of growth. The MIC was taken as the lowest concentration that will prevent the growth of the test bacterial isolates.

### 4.9. Determination of Minimum Bactericidal Concentrations of the Extract and Fractions of P. americana

The MBCs were determined using the Olorundare [23] method with little modification. Samples were taken from plates with no visible growth in the MIC assay and sub-cultured onto freshly-prepared nutrient agar plates and later incubated at 37 °C for 72 h. The MBC was taken as the concentration of the extract that did not show any bacterial growth on fresh agar plates.

### 4.10. Determination of the Rate of Killing of the Test Isolate Using the Butanolic Fraction

The assay of the rate of killing of the test isolate was determined in accordance with the method of Odenholt [24] with little modification. The turbidity of an 18 h old broth culture of the test organism was standardized to approximately 10^6^ cfu/mL. An amount of 0.5 mL of the standardized suspension was added to 4.5 mL of different concentrations of the fraction relative to the MIC. These were held at room temperature, and the killing rate was determined over a period of 2 h. Exactly a 0.5 mL volume of each suspension was withdrawn at time intervals and transferred to 4.5 mL of nutrient broth recovery medium containing 3% “Tween 80” to neutralize the effect of the antimicrobial compound carry-overs from the test organisms. The suspension was then serially diluted and plated for viable counts. The plates were later incubated at 37 °C for 48 h. The control plates contained the test cells without the extract. The emergent bacterial colonies were counted and compared with the counts of the culture control.

### 4.11. Determination of Protein Leakage from the Test Organisms

Eighteen hour old test cells were washed three times in physiological saline by centrifugation at 10,000 rpm for 10 min followed by re-suspension in physiological saline. Washed suspension test cells were standardized and later treated with various concentrations of the butanolic fraction relative to the MIC at various time intervals for a period of 2 h. Each suspension was centrifuged at 7000 rpm, and the supernatant obtained was assayed for protein using the Bradford [25] method. The concentration of protein was estimated from the established standard curve obtained using bovine serum albumin (BSA).

### 4.12. Determination of Potassium Ion Leakage from the Test Organisms

Fifty milliliters of harvested and washed cells (OD_470 nm_ = 1.5) were placed in a clean 100 mL beaker, which was magnetically stirred. A volume (5 mL) of ionic strength adjustment buffer (ISAB; 18.37 g of tetraethylammonium chloride in deionized water and made up to 100 mL) was added to the beaker. This ensured that the background ionic strength of all solutions was kept constant. The potassium ion sensing electrode (Qualiprobe QSE 314, EDT Instruments Waldershare Park, Dover, UK) and its reference electrode (Qualiprobe double junction reference electrode E8092, EDT Instruments) were placed into the cell suspension. The potential difference (mV) derived by the electrodes was measured using a Whatmann PHA 220 pH/mV meter (Whatmann, Maidstone, UK). Bacterial cells were treated with various concentrations of the butanolic fraction of the plant extract relative to the MIC. The potassium efflux from the cells in the suspension was measured at time intervals over 2 h as a potential difference in mV. These values were converted to concentrations of K^+^ ions (M) by reference to a conversion graph, which had been constructed earlier using KCl standard solutions. The concentration of K^+^ ions released was plotted against time.

## 5. Conclusions

The stem bark of *Persea americana* could be a potential source of antibacterial compounds of natural origin that can be used in producing drugs to treat foodborne infection caused by *Bacillus cereus* and other pathogens. The probable mechanism of action of this plant extract may be by way of cell membrane disruption of the *B. cereus* cells used in our findings, although there might be other mechanism(s) of action exerted by this extract leading to the cell death.

This present study investigates the *in vitro* antibacterial potentials of stem bark extract and butanolic fraction of *Persea americana* at concentrations of 25 mg/mL and 10 mg/ml respectively, on strains of *Bacillus cereus* implicated in food poisoning. Efforts are ongoing in our laboratories to carry out the *in vivo* studies in animal models and to assess the cytotoxity level of the plant. Also, the isolation of bioactive compounds with accompany detailed structural elucidation of the isolated compound(s) responsible for the activities reported in this study is the focus of our further research.
